# Hybrid 3D Printed and Electrospun Multi-Scale Hierarchical Polycaprolactone Scaffolds to Induce Bone Differentiation

**DOI:** 10.3390/pharmaceutics14122843

**Published:** 2022-12-19

**Authors:** Ainhoa Gonzalez-Pujana, Teresa Carranza, Edorta Santos-Vizcaino, Manoli Igartua, Pedro Guerrero, Rosa Maria Hernandez, Koro de la Caba

**Affiliations:** 1 NanoBioCel Research Group, Laboratory of Pharmaceutics, School of Pharmacy, University of the Basque Country (UPV-EHU), 01006 Vitoria-Gasteiz, Spain; 2 Biomedical Research Networking Centre in Bioengineering, Biomaterials and Nanomedicine (CIBER-BBN), Institute of Health Carlos III, 28029 Madrid, Spain; 3 Bioaraba, NanoBioCel Research Group, 01006 Vitoria-Gasteiz, Spain; 4 BIOMAT Research Group, University of the Basque Country (UPV/EHU), Escuela de Ingeniería de Gipuzkoa, Plaza de Europa 1, 20018 Donostia-San Sebastián, Spain; 5 Domotek SL, B° Santa Luzia 17, 20400 Tolosa, Spain; 6 BCMaterials, Basque Center for Materials, Applications and Nanostructures, UPV/EHU Science Park, 48940 Leioa, Spain; 7 Proteinmat Materials SL, Avenida de Tolosa 72, 20018 Donostia-San Sebastián, Spain

**Keywords:** 3D printing, electrospinning, bone regeneration

## Abstract

Complex scaffolds composed of micro- and nano-structures are a key target in tissue engineering and the combination of sequential 3D printing and electrospinning enables the fabrication of these multi-scale structures. In this work, dual 3D printed and electrospun polycaprolactone (PCL) scaffolds with multiple mesh layers were successfully prepared. The scaffold macro- and micro-porosity were assessed by optical and scanning electron microscopy, showing that electrospun fibers formed aligned meshes within the pores of the scaffold. Consequently, the hydrophilicity of the scaffold increased with time, enhancing cell adhesion and growth. Additionally, compression tests in back and forth cycles demonstrated a good shape recovery behavior of the scaffolds. Biological results indicated that hybrid PCL scaffolds are biocompatible and enable a correct cell culture over time. Moreover, MC3T3-E1 preosteoblast culture on the scaffolds promoted the mineralization, increased the alkaline phosphatase (ALP) activity and upregulated the expression of early and late osteogenic markers, namely ALP and osteopontin (OPN), respectively. These results demonstrate that the sequential combination of 3D printing and electrospinning provides a facile method of incorporating fibers within a 3D printed scaffold, becoming a promising approach towards multi-scale hierarchical scaffolds capable of guiding the osteogenic differentiation.

## 1. Introduction

The development of new biomaterials that can contain cells or growth factors is a key issue in tissue engineering to mimic the native structure of tissues and organs. In this field, additive manufacturing has been an advance, not only for the manufacturing industry but also for regenerative medicine [1,2]. Thanks to the ability to create custom structures with repetitive and specific internal morphology, a very wide field to provide custom solutions for the creation of scaffolds for the regeneration of tissues such as skin, cartilage or bone has been opened up [3,4,5].

The most important factor determining a good behavior of the scaffold is the biomimetic capacity of the target tissue environment [6]. In the case of bone tissue regeneration, the complexity lies in the shape of the structure, internal porosity, degradability, and mechanical properties [3,7]. Regarding the internal structure, the porous hierarchy of this tissue is a relevant issue, since this influences the penetration and distribution of cells, allows the transport of gas and nutrients, and ensures good cell adhesion and spreading [5,8,9,10]. Pore sizes of 300 µm are the most promising to ensure new bone formation, while 100 µm pore size is considered the minimum to achieve cell migration and nutrient and gas transport [11].

Many authors have proposed electrospinning technology as a possible solution for this purpose, which offers the opportunity to introduce nanoscale fibers that reproduce the extracellular matrix (ECM) [12,13,14]. This technique consists of generating nanofibers using a potential difference so that the surface tension of a polymer in solution forms a drop that can be stretched towards the electric field and, thus, forms a fiber by the evaporation of the solvent. It is a simple process where parameters such as the dielectric constant of the solvent, the flow rate of the polymer solution, the applied voltage differential, and the distance for the jet to surge and form nanofibers take part [15].

This technology can achieve this microenvironment, but resulting scaffolds usually show poor mechanical properties, which make it insufficient on its own [14,16,17]. The combination of two technologies, 3D printing and electrospinning, has been proposed to provide an integral solution for the problem of biomimetic porous architectures of bone tissue. The 3D network formed by printed microfibers and electrospun nanofibers is a promising approach to obtain mechanically stable structures while maintaining the interconnectivity between fibers and providing adequate porosity for cell development. However, previous studies incorporating electrospinning to 3D printing have spun yarn only on the top surface of the scaffold, producing very dense meshes that limited cell migration and infiltration, which is a common problem with electrospinning. In contrast, this work does not propose the fabrication of a bilayer by 3D printing and electrospinning, but employs both technologies sequentially to produce a fully integrated hierarchical structure through the different layers.

The use of polycaprolactone (PCL), an aliphatic biocompatible polyester, has been proposed for this work. Among its most interesting features are the slow biodegradability, good mechanical properties and non-toxic degradation products, which are easily eliminated by the human metabolic system; moreover, the biomaterial has been approved by the US Food and Drug Administration (FDA) [7,12,18,19]. In the present study, dual 3D printed and electrospun PCL scaffolds were fabricated. The resulting hybrid PCL scaffolds were characterized and their biocompatibility and suitability for cell culture were assessed. Moreover, the ability of these novel scaffolds to guide the osteogenic differentiation was evaluated in primary murine MC3T3-E1 preosteoblasts.

## 2. Materials and Methods

### 2.1. Materials

PCL (PBI-010) pellets were purchased from Natureplast (Ifs, France). PCL FacilanTM filament was purchased from 3D4makers (Haarlem, The Netherlands). Acetone and DMSO were purchased from Mezcla Perfecta (Madrid, Spain). The 10 mL polypropylene syringes, needles and nozzles were purchased from Domotek (Gipuzkoa, Spain).

### 2.2. Preparation of Hybrid 3D Scaffolds

**Figure 1:**
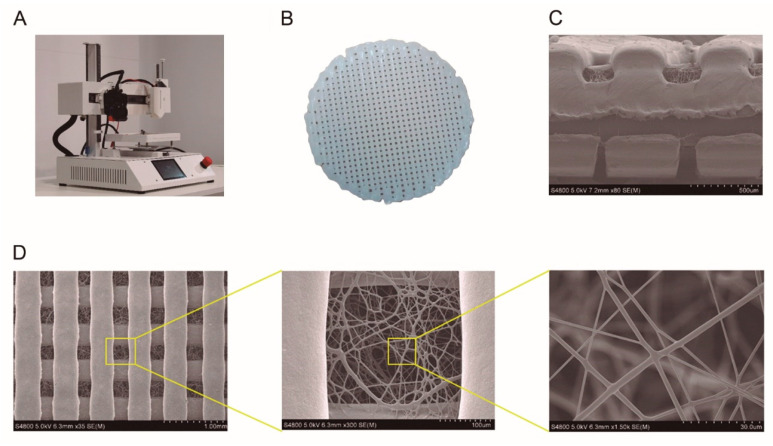
**Hybrid PCL scaffolds.** (**A**) Domotek domoBIO 2A bioprinter, equipped with an electrospinning module and a filament extruder, used to manufacture the scaffolds. (**B**) Macroscopic image of a hybrid PCL scaffold. (**C**) SEM image of the scaffold cross section; scale bar: 500 µm. (**D**) SEM image of the scaffold surface; scale bar from left to right hand: 1.00 mm, 100 µm, and 30.0 µm.

To prepare a 12 wt% PCL solution for electrospinning, PCL was dissolved by magnetic stirring for 1 h at 40 °C in acetone/DMSO binary dissolvent system (weight ratio 80:20). The hybrid PCL scaffolds were fabricated using a domoBIO 2A bioprinter (Domotek, Gipuzkoa, Spain), equipped with an electrospinning module, filament extruder and high-performance heated platform (Figure 1A). Solid Edge (Siemens, Munich, Germany) software was used to create printable designs: 21 mm of diameter and 1 mm of height discs. Simplify 3D software (Simplify3D, Cincinnati, OH, USA) was used for slicing all the stages. Processing parameters are detailed in Table 1.

Hybrid PCL scaffolds were composed of successive layers of 3D printed PCL filament and electrospun PCL solution, with a total of 10 layers, forming 21 mm diameter and 1 mm high discs. Electrospinning was carried out at 5 mm/s above the collector on the 3D printed layer. Regarding the height of the final object, it is worth noting that the 3D printed layer had a height of 0.2 mm, while the electrospun layer had a height of less than 100 µm; therefore, 3D printing was the technique that determined the height of the final object.

Electrospinning solution was extruded by 10 mL syringe with 22 G needle at 0.15 mL/min at total fiber deposition time of 1 min. During this stage, the applied voltage was 11 kV and the aluminum collector with fabricated lines was maintained at 9 cm. The driving forces of the electrospinning stage include the flow rate and the distance between the point where the high voltage is applied and the grounding platform. These two variables were kept constant throughout the manufacturing process and, thus, they did not affect the outcome of the final construction.

Filament was 3D printed with a 1.75 mm diameter polymer filament and 0.4 mm brass nozzle. The extruder and bed temperatures were 110 °C and 50 °C, respectively. This tool head made a 21 mm diameter cylinder at 30 mm/s global speed, with 0.2 mm layer height and 75% infill percentage without perimeter line.

All scaffolds were kept at 40 °C for 10 min on the platform to ensure that no solvent remained. At least 100 hybrid scaffold units (Figure 1B) were prepared for testing. It should be noted that the resulting scaffolds were homogeneous and repetitive; therefore, identical results were achieved in the characterization of the scaffolds manufactured in different fabrication series.

### 2.3. Scaffold Characterization

Fourier transform infrared (FTIR) spectra were obtained using spectrometer Alplha II plus (Bruker, Barcelona, Spain), equipped with a ZnSe ATR cristal accessory. Thirty two scans were performed at 4 cm^−1^ resolution, and all the samples were analyzed in the spectral range of 4000–700 cm^−1^.

Differential scanning calorimetry (DSC) was performed from −10 °C to 250 °C at 10 °C/min under nitrogen atmosphere with Mettler Toledo DSC 822 (Mettler Toledo, Barcelona, Spain).

Thermo-gravimetric analysis (TGA) was carried out using a SDTA 851 TGA (Mettler Toledo, Barcelona, Spain). Experiments were performed from 25 to 800 °C at 10 °C/min under nitrogen atmosphere.

X-ray diffraction (XRD) measurements were performed by a diffraction unit PANalytical Xpert PRO (PANalytical, Almelo, The Netherlands). The radiation was generated from a Cu-Kα (λ = 1.5418 Å) source operating at 40 kV and 40 mA. The diffraction data were collected from 2θ values from 2° to 50°.

A Hitachi S-4800 scanning electron microscope (Hitachi High-Technologies Corporation, Tokyo, Japan) with an acceleration voltage of 15 kV was used to visualize the scaffold morphologies. Scaffolds were placed on a metal stub and coated with gold using a JEOL fine-coat ion sputter JFC-1100 (JEOL Ltd., Tokyo, Japan) under argon atmosphere.

The optical contact angle measurement and drop contour analysis were carried out by the OCA 20 system from Dataphysics (Neurtek, Eibar, Spain). Scaffolds were placed on a moving stage and 3 μL of distilled water was placed on the scaffold surface to estimate the hydrophilic character at room temperature.

Compression tests were performed using a TA.XT.Plus C Texture Analyzer (Aname Instrumentación Científica, Madrid, Spain), equipped with a 50 kg load cell. The analysis was carried out using an aluminum cylinder of 50 mm diameter (P/50), crosshead speed was set at 1 mm/s and the activation force was 5 g. Cyclic compression sweeps were carried out up to 80% strain. The software used for the analysis was Texture Exponent 32.

### 2.4. Biocompatibility of Hybrid PCL Scaffolds

To determine the biocompatibility of hybrid PCL scaffolds, cytotoxicity studies compliant with the ISO 10993-5:2009 guidelines—intended for the biological evaluation of medical devices—were performed. Following these guidelines, L-929 fibroblasts (ATCC) were employed. For their culture, Eagle’s Minimum Essential Medium (ATCC) was used, supplemented with 10% (*v*/*v*) inactivated horse serum and 1% (*v*/*v*) penicillin–streptomycin. Cells were maintained at 37 °C in a 5% CO_2_/95% air atmosphere and passaged every 2–3 days at 70–90% confluence. For all experiments, cells were used at passages 4–5.

For indirect cytotoxicity studies, L-929 fibroblasts were seeded on 96-well plates—at a density of 5000 cells/well—and overnight cultured to allow cell attachment. Subsequently, the culture medium was replaced by hybrid PCL extraction medium. To obtain this extraction medium, hybrid PCL scaffolds were UV-sterilized (10 min of UV exposure to each side of the scaffold) and incubated in complete EMEM medium for 24 h at 37 °C in agitation. For the blank control group, L-929 cells were cultured with regular complete EMEM medium. After 48 h of incubation with the extraction medium, cell viability was evaluated by means of a Cell Counting Kit-8 (CCK-8) colorimetric assay (Merck). In brief, cells were washed with PBS *w*/*o* Ca^+2^ and Mg^+2^ (Gibco, Madrid, Spain) and incubated for 4 h at 37 °C in complete EMEM supplemented with 10% CCK-8 reagent. For the positive control group, the medium was additionally supplemented with dimethyl sulfoxide (DMSO). After incubation, plates were read at 450 nm with reference wavelength at 650 nm (Infinite M200 TECAN, GMI Inc., Madrid, Spain).

For direct cytotoxicity studies, L-929 fibroblasts were seeded on 12-well plates—at a density of 70,000 cells/well—and overnight cultured to allow cell attachment. Subsequently, a hybrid PCL scaffold was placed in each well. For the blank control group, scaffolds were not included in the wells. Cells were then maintained in culture for additional 48 h, when the CCK-8 assay was employed to determine cell viability.

### 2.5. MC3T3-E1 Culture in Hybrid PCL Scaffolds

Primary murine MC3T3-E1 preosteoblasts were cultured in MEM-α *w*/*o* ascorbic acid (Gibco, Madrid, Spain) supplemented with 10% (*v*/*v*) fetal bovine serum (FBS) and 1% (*v*/*v*) penicillin–streptomycin. Cells were maintained at 37 °C in a 5% CO_2_/95% air atmosphere and passaged every 2–3 days, when reaching 70–90% confluence. For all experiments, cells were used at passages 6–8.

To optimize cell attachment, each side of the hybrid PCL scaffolds was treated with oxygen plasma (Harrick Plasma cleaner, PDC-002) for 1 min at an oxygen pressure of 0.7 bar and a high power. Afterwards, scaffolds were sterilized by exposing each side to UV-light for 10 min in a laminar flow hood. The ready-to-use Hybrid PCL scaffolds were then casted in 12-well non-treated plates (Thermo Scientific, Madrid, Spain).

MC3T3-E1 preosteoblasts were detached from culture (trypsin-EDTA, Gibco, Madrid, Spain) and suspended in FBS free MEM-α at a density of 500,000 cells/mL. A total of 1 mL of the resulting suspension was transferred to each well of the culture plates containing the scaffolds. Plates were incubated in agitation for 2 h at 37 °C. Subsequently, to eliminate the non-attached cell excess, the scaffolds were transferred to clean 12-well non-treated plates, with fresh complete MEM-α, and maintained at 37 °C in a 5% CO_2_/95% air atmosphere for the cell studies.

### 2.6. MC3T3-E1 Morphology and Distribution on Hybrid PCL Scaffolds

The distribution and morphology of MC3T3-E1 cells cultured on hybrid PCL scaffolds were monitored over 21 days. At different time points, the scaffolds were washed with PBS *w*/*o* Ca^+2^ and Mg^+2^ (Gibco, Madrid, Spain) and fixed with 4% (*w*/*v*) formaldehyde (Panreac, Barcelona, Spain) for 10 min. Afterwards, samples were permeabilized with 1X triton (Merck, Madrid, Spain) for 5 min. Cells were stained with Alexa Fluor 488-labelled phalloidin for B-actin (Fisher Scientific, Madrid, Spain) and DAPI for the nuclei (Fisher Scientific, Madrid, Spain). Z-stack images were obtained by means of an inverted microscope equipped with a DSD2 confocal modulus (Nikon TMS, Barcelona, Spain).

### 2.7. Viability and Proliferation of MC3T3-E1 Cells in Hybrid PCL Scaffolds

The viability and proliferation of MC3T3-E1 cultured on hybrid PCL scaffolds were monitored over a course of 21 days. At different time points, scaffolds were washed with PBS *w*/*o* Ca^+2^ and Mg^+2^ (Gibco, Madrid, Spain) and transferred to new non-treated 12-well plates. For the determination of metabolic activity, 1 mL of complete MEM-α *w*/*o* ascorbic acid (Gibco, Madrid, Spain) supplemented with 10% CCK-8 reagent was added to each well. After 4 h of incubation at 37 °C, absorbance was read at 450 nm—with reference wavelength at 650 nm—(Infinite M200 TECAN, GMI Inc., Madrid, Span). To determine cell proliferation, cells were detached from hybrid PCL scaffolds by means of trypsin-EDTA (Lonza, Pontevedra, Spain). Cell number was determined using a benchtop TC20 Automated Cell Counter (Bio Rad, Madrid, Spain).

### 2.8. Mineralization of MC3T3-E1 Cells

MC3T3-E1 mineralization was monitored over 21 days by means of Alizarin Red S staining. Scaffolds were washed with PBS *w*/*o* Ca^+2^ and Mg^+2^ (Gibco, Madrid, Spain) and transferred to new non-treated 12-well plates. Cells were fixed with 4% (*w*/*v*) formaldehyde (Panreach, Barcelona, Spain) for 10 min. After 3 washings with ddH_2_O water, scaffolds were immersed in a 1 mg/mL solution of Alizarin Red S (Merck) for 30 min. Subsequently, scaffolds were washed 3 times with ddH_2_O water. The final washing was maintained in agitation for 1 h to ensure complete clearance of the Alizarin Red S excess. Images of the stained scaffolds were acquired using a Optika SZP 10 microscope equipped with a Nikon DS-Fi3 color camera.

Afterwards, scaffolds were incubated for 1 h with 6 M HCl to extract the Alizarin Red S. Absorbance was measured at 570 nm and results were quantified by means of a standard curve.

### 2.9. Intracellular Alkaline Phosphatase (ALP) Activity Determination

The intracellular ALP activity of MC3T3-E1 cells cultured in hybrid PCL scaffolds was monitored during 21 days. At different time points, cells were detached from the scaffolds using trypsin-EDTA (Lonza) and counted by means of the TC20 Automated Cell Counter (Bio-Rad, Madrid, Spain) to normalize the obtained results. Cells were resuspended in 0.5 mL of ddH_2_O water and incubated at 37 °C for 1 h to enable cell lysis, with the subsequent release of intracellular ALP. A control group was included by repeating this procedure with MC3T3-E1 cells 2D cultured for the same time period.

ALP activity was determined by analyzing its capacity to hydrolyze p-nitrophenyl phosphate (pNPP) at pH = 9.3. Briefly, cell lysates were added to a 96-well plate. In the same plate, a standard curve was included using calf intestinal ALP. Subsequently, 100 μL of 0.2% (*w*/*v*) pNPP were added to each sample. After 30 min, the reaction was stopped by adding 50 μL of 3 M NaOH and absorbance was read at 405 nm (Infinite M200 TECAN, GMI Inc., Madrid, Spain).

### 2.10. RNA Isolation and Reverse Transcription

At different time points MC3T3-E1 cells were detached from hybrid PCL scaffolds using trypsin-EDTA (Lonza). Retrieved cells were counted (TC20 Automated Cell Counter, Bio Rad, Madrid, Spain) and centrifuged to obtain a cell pellet.

RNA isolation and purification were performed by means of PureLink RNA Micro Kit (Invitrogen) following the manufacturer’s indications. The lysis buffer provided with the kit was supplemented with 1% β-mercaptoethanol (Merck, Madrid, Spain) and 350 µL of the resulting solution were added to each cell pellet. To eliminate DNA contaminations, an additional step was included in the process by performing DNAse treatment (Purelink DNAse set, Invitrogen, Madrid, Spain). To elute RNA, 30 μL of nuclease-free water were employed. RNA yield and quality were assessed by means of the SimpliNano™ Spectrophotometer (Biochrom, Granada, Spain). RNA samples were stored at −80 °C until further use.

For RNA reverse-transcription, the T100 Thermal cycler (BioRad, Madrid, Spain) was employed. Briefly, 5 µL of sample were mixed with 15 µL of iScript Advanced Reverse Transcription Supermix for real-time RT-qPCR (Bio-Rad, Madrid, Spain). The reaction was carried out as indicated in manufacturer instructions: 46 °C for 20 min and 95 °C for 1 min. cDNA was stored at −80 °C until real-time RT-qPCR analyses.

### 2.11. Real-Time RT-qPCR

For real-time RT-qPCR analyses, 10 ng of cDNA from each sample were mixed with 2 × AdvancedSSO SYBR Green Supermix (Bio-Rad, Madrid, Spain) and primers (PrimePCR primers, BioRad, Madrid, Spain) a total volume of 20 μL. Individual gene primers are detailed in Table S1.

Each sample was loaded in duplicate in MicroAmp Fast Optical 96-Well Reaction Plates (Applied Biosystems, Madrid, Spain) and run on a StepOnePlus™ Real-Time PCR System (Applied Biosystems, Madrid, Spain) following the protocol: 2 min at 50 °C, 2 min at 95 °C, (15 s at 95 °C and 1 min at 60 °C) × 40 cycles. In addition to samples, non-template (NTC) and non-reverse transcription (NRT) controls were included, in which no amplification was detected. A dilution series of the starting cDNA was also included in the plates to evaluate primer efficiency (E). In particular, the slope of a linear regression of the Cq values obtained from the dilution series was used to calculate E:

(1) $$E=10^{\left(-\frac{1}{\mathrm{slope}}\right)}$$

Moreover, melt curves were also evaluated to confirm the single-product amplification. Relative gene expression was calculated via the Livak method, using *Gapdh* as reference gene. Error was propagated as follows:

(2) $$\mathrm{Error}\;\left(a+b\right)=\sqrt{\mathrm{Error}\;{\left(a\right)}^{2}+\;\mathrm{Error}\;{\left(b\right)}^{2}}$$

### 2.12. Data Analysis and Statistics

The Shapiro–Wilk test was performed to evaluate the normal distribution of the data. For 2-group comparisons, when data followed a normal distribution, Student’s *t*-test was employed, whereas in the case of non-normally distributed data the non-parametric Mann–Whitney U test was performed. For multiple comparisons, normally distributed data were analyzed by means of one-way ANOVA, evaluating the homogeneity of variances with the Levene’s test. When variances were homogeneous, the Bonferroni test was chosen as a post-hoc. In case of non-homogeneous variances, the Tamhane post-hoc test was selected. For multiple comparisons on non-normally distributed data, the Kruskall–Wallis test was employed. Statistical analyses for RT-qPCR data were carried out with the delta-Ct values. For all analyses, *p*-values lower than 0.05 were assumed to be significant. SPSS 23 was employed to perform all statistical computations (IBM SPSS, Chicago, IL, USA).

## 3. Results and Discussion

All samples showed a similar appearance and a homogeneous mass distribution (CV = 1.71%), as can be seen in Figure S1. Three-dimensional printed lines were separated by 300 µm distance and nanofibrous mat filled this space (Figure 1C). The electrospun fibers acted as bridges between the printed lines, and fibers were homogeneously distributed, building a porous network with an average porosity of 20.2 ± 6.0 µm. As observed by optical microscopy (Figure S2), nanofibrous mat was defect-free, very smooth, and very well integrated into 3D printed lines, leading to a very porous structure. In addition, the technology used allows the collection of electrospun fibers in different directions (Figure 1D).

SEM analysis confirmed that 3D printed layers were well deposited and nanofibrous mat was well-integrated, making a sandwich-type structure. No beads or droplets were observed in the electrospun fibers, indicating that the processing conditions were optimal. It is worth noting that the electrospun mat was not only deposited on the surface of each 3D printed line but also interconnected with other mats, as shown in the cross-sectional view of the scaffolds (Figure 1C), indicating that the material (PCL filament and PCL solution) deposited in the different processing stages was well joined. In addition, mat layers filled the pores between the 3D printed layers. This type of structure is an approach to make porous scaffolds with interconnected macrochannels, which play an important role in bone regeneration to stimulate the vascularization and osteogenesis, and interconnected microchannels, which enhance osteochondral ossification [10]. The ECM of most tissues has an anisotropic architecture, so the fabrication of fibers with optimal density is a keypoint to mimic the native structure and has a positive effect on cell adhesion and tissue regeneration.

**Figure 2:**
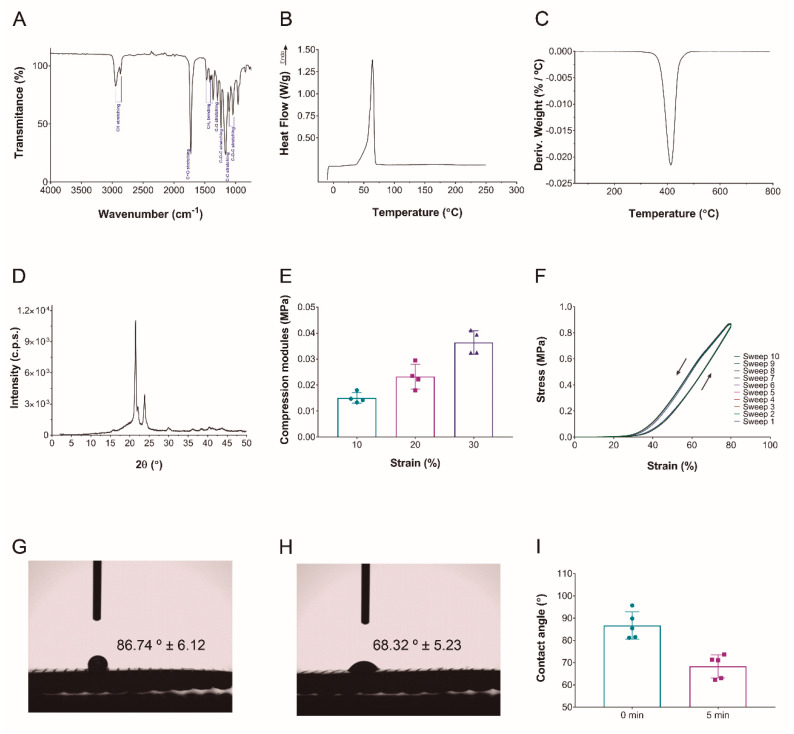
**Hybrid PCL scaffold characterization.** (**A**) FTIR spectrum. (**B**) DSC curve. (**C**) DTGA curve. (**D**) XRD pattern. (**E**) Compression module values calculated at different strain values. (**F**) Stress-strain curves of a ten compression-decompression cycle. (**G**) Water contact angle at initial time. (**H**) Water contact angle at 5 min. (**I**) Mean value and standard deviation of contact angle values at 0 and 5 min.

FTIR spectroscopy was performed to assess whether there was any change due to the processing conditions employed. FTIR spectrum showed two bands at 2945 cm^−1^ and 2860 cm^−1^ corresponding to C-H stretching of asymmetric and symmetric groups. The signal at 1724 cm^−1^ was attributed to C=O stretching, whereas the band at 1294 cm^−1^ and the sharp one at 1155 cm^−1^ were assigned to C-O and C-C stretching vibrations of crystalline and amorphous phases. Finally, the bands at 1236, 1105 and 1043 cm^−1^ correspond to asymmetric C-O-C stretching, while the bands at 1469, 1398 and 1363 were related to CH_2_ bending. The spectrum shown in Figure 2A was the typical FTIR spectrum for PCL, with no evidence of reaction due to the processing conditions used [20,21].

DSC analysis was carried out to assess whether there was remaining solvent in the scaffolds developed. As shown in Figure 2B, only one peak was observed, associated to the melting temperature of PCL at 64.17 °C, with a melting enthalpy of 64.04 mJ g^−1^. Similar values have been reported in literature [22,23,24]. In the same manner, TGA was performed to confirm the absence of solvent in the scaffolds. As can be seen in Figure 2C, only one peak was observed at 413 °C, related to the degradation of the polymer [25].

Additionally, XRD analysis (Figure 2D) showed the semicrystalline nature of the scaffolds developed. Peaks at 21.5° and 23.8° revealed (110) and (200) crystallinity reflections, respectively, representing orthorhombic crystal structure [22,23,24]. Therefore, both DSC and XRD analyses confirmed that PCL used to fabricate hybrid scaffolds was semicrystalline. As reported by Camarero-Espinosa, the greater crystallinity of the scaffolds means a better biocompatibility due to their capacity to maintain their integrity for longer until the degradation process occurs, limiting immunologic response [26]. In the case of bone tissues, the time needed to promote new tissue formation is very important, because they are very slow formation tissues, so the scaffold should degrade in a more controlled manner [4,10,27,28].

Since 3D printed structures often exhibit anisotropic mechanical properties due to changes in weld quality, the mechanical behavior in compression and cyclic loading regimes was evaluated. Compression under loading and unloading cycles was carried out to obtain stress at maximum strain of 80%. Only elastic deformation was shown in Figure 2E; the cyclic strains stress under loading curve showed that the material had good mechanical absorption capacity until 30% of strain, although it started changing this property at this point. No plastic deformation was observed in any stress–strain curves [28]. Scaffolds showed good shape recovering behavior after 10 compression sweeps (Figure 2F). These values were determined by 3D printed fibers and not by electrospun mats [4]. Although the modulus values obtained were far from the range of real bone modulus values, the observed shape recovery was a desirable property for bone tissue engineering and supposes an approximation to the real microenvironment of these type of tissues [4,28,29,30].

As reported in the literature, water contact angle values (86 ± 6°) showed that hybrid scaffolds had hydrophobic nature (Figure 2G) [24,31]. After 5 min, the water drop was partially absorbed into the scaffold, as shown in Figure 2H, confirming that the scaffold structure is porous enough for cells. This promotes nutrient and water diffusion, in contrast to very dense meshes that limited cell migration and infiltration, which is a common problem with electrospinning that can be crucial in the development of functional tissues. In this work, the density of the mesh obtained was analyzed by measuring the hydrophobicity of the scaffolds to assess their suitability for cell culture studies. Based on the spacing of the fibers and the decrease in hydrophobicity of the scaffolds (Figure 2I), results indicated that the structure obtained is neither too dense to maintain hydrophobicity nor too porous to hinder cell adhesion [24,32,33].

**Figure 3:**
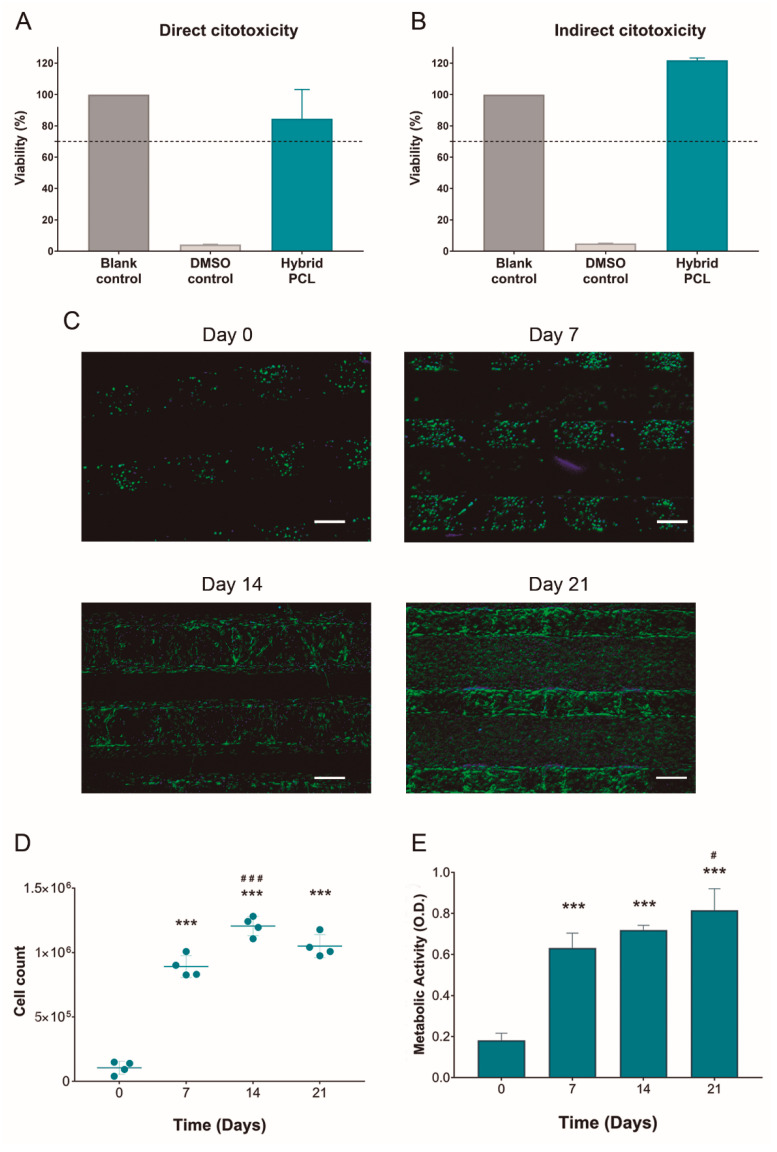
**Cell culture on hybrid PCL scaffolds.** (**A**) Direct and (**B**) indirect cytotoxicity studies performed with L-929 fibroblasts following ISO 10993-5:2009 guidelines. (**C**–**E**) MC-3T3 murine osteoblast culture on hybrid PCL scaffolds: (**C**) fluorescent DAPI/Phalloidin micrographs for cell distribution and morphology, (**D**) proliferation and (**E**) metabolic activity of MC-3T3 at different time points. Results are shown as the mean ± standard deviation (*n* = 4). Scale bars = 200 μm. Statistical significance: *** *p* < 0.001 compared to day 0. # *p* < 0.05 and ### *p* < 0.001 compared to day 7.

Considering that biomedical materials must present an adequate biocompatibility, in vitro cytotoxicity studies compliant with the ISO 10993-5:2009 guidelines for biomedical devices were performed. For direct cytotoxicity assays, L-929 fibroblasts were cultured in direct contact with the hybrid PCL scaffolds for 48 h (Figure 3A). In the case of the indirect cytotoxicity test, the cells were cultured for 48 h with extraction medium—obtained from incubating hybrid PCL scaffolds in complete culture medium for 24 h (Figure 3B). The variability observed between viability values in direct and indirect cytotoxicity tests was due to the inherent experimental differences between both assays, as also observed in other works [34,35]. Indeed, a recent publication highlighted that the differences between both assays makes the assessment by both methods necessary [35]. In both tests, the cells cultured in hybrid PCL scaffolds scored viability values above the 70% of the blank controls—which were 2D cultured in plates with regular complete culture medium. Therefore, hybrid PCL scaffolds can be considered as non-cytotoxic biomaterials according to the ISO 10993-5:2009 guidelines.

Once demonstrated biocompatible, the suitability of hybrid PCL scaffolds to culture MC3T3-E1 preosteoblasts was evaluated. With that aim, MC3T3-E1 cultured on the scaffolds were monitored over the course of 21 days. Regarding cell distribution and morphology, DAPI/Phalloidin-staining showed an increase in both cell spreading and cell number over time (Figure 3C and Figure S3). Such proliferation was corroborated with the cell counts at different time points (Figure 3D), which showed a statistically significant increase by day 7, in comparison to the initially seeded cell numbers. From day 7 on, proliferation was not so notable, probably due to the spacing limitations in the scaffolds, as observed in the DAPI/Phalloidin images. In this line, the metabolic activity of MC3T3-E1 cells followed a similar pattern (Figure 3E). As the observations in Figure 2G–H indicated, these results confirm that the structure of hybrid PCL scaffolds present an adequate density that allows not only primary cell attachment but also the stabilization of the culture overtime, with cells spreading and migrating.

**Figure 4:**
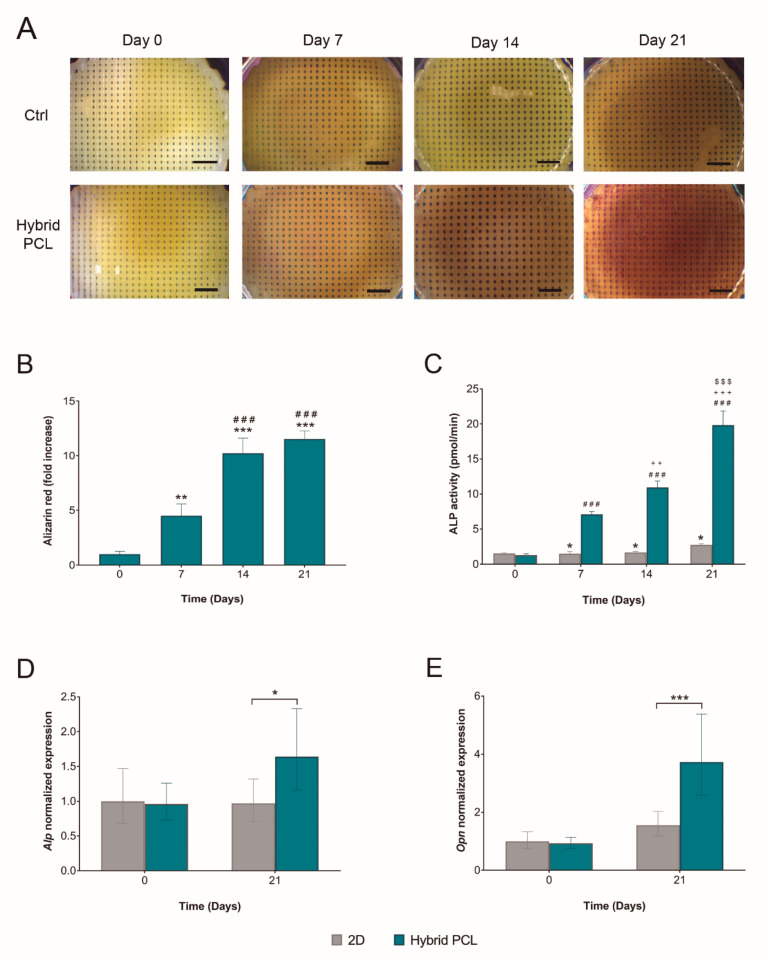
**Osteogenic differentiation of MC3T3-E1 cells cultured on hybrid PCL scaffolds.** (**A**) Optical images of calcium deposits stained with Alizarin Red S. Scale bars = 2.5 mm. (**B**) Quantification of the calcium deposits stained with Alizarin Red S. Statistical significance: ** *p* < 0.01, *** *p* < 0.001 compared to day 0. ### *p* < 0.001 compared to day 7. (**C**) Overtime intracellular ALP activity determination in MC3T3-E1 cells cultured either on hybrid PCL scaffolds or on regular culture plates (2D). Statistical significance: * *p* < 0.05 compared to cells cultured on hybrid PCL scaffolds at that same time point. ### *p* < 0.001 compared to cells cultured on hybrid PCL at day 0. +++ *p* < 0.001, ++ *p* < 0.01 compared to cells cultured on Hybrid PCL at day 7. $$$ *p* < 0.001 compared to cells cultured on hybrid PCL at day 14. (**D**) *Alp* and (**E**) *Opn* expression in MC3T3-E1 cells cultured either on hybrid PCL scaffolds or on regular culture plates (2D), all normalized to 1 as fold increase of day 0. *Gapdh* was used as reference gene. Statistical significance: * *p* < 0.05, *** *p* < 0.001. Results are shown as the mean ± standard deviation in (**A**,**B**), and as mean ± S.E in (**C**,**D**), (*n* = 4).

Consequently, the next step was to assess if hybrid PCL scaffolds were able to promote the osteogenic differentiation of MC3T3-E1 preosteoblasts. With that aim, we first studied the mineral deposition of these cells. The accumulation of calcium deposits has been correlated to osteogenesis and, therefore, considered a marker for bone regeneration. Here, Alizarin Red S staining was performed to qualitatively evaluate the calcium deposition [36]. Optical images in Figure 4A show an overtime increase in the calcium levels deposited by MC3T3-E1 cells cultured on hybrid PCL scaffolds. To quantify these results, Alizarin Red S staining can be extracted and spectrophotometrically determined [37]. In the present study, Alizarin Red S was extracted with 0.6 M HCl (Figure 4B). The colorimetric measurements supported the optical observations, demonstrating a statistically significant increase in calcium deposits until day 14, the point from which thereon the deposition stabilized.

To study the osteogenic capacity of MC3T3-E1 preosteoblasts at the molecular level, the intracellular ALP activity was measured. ALP is expressed ubiquitously in bone-forming cells, and it is directly involved in early osteogenesis and in the hydrolysis of multiple types of phosphates, thus, enhancing cell maturation and calcification [38]. Here, ALP activity was analyzed over a course of 21 days in cells cultured either on hybrid PCL scaffolds or 2D on regular culture plates (Figure 4C). Results showed that from day 7 on, cells cultured on the scaffolds presented a significantly increased intracellular ALP activity compared to the 2D control. Moreover, a progressive increase was observed when comparing the ALP activity of MC3T3-E1 preosteoblasts cultured on Hybrid PCL scaffolds over time.

To further explore these findings, the gene expression of ALP and OPN at the initial and final points of the time course was analyzed next (Figure 4D,E). These genes were selected for being makers of different stages of osteogenesis [39]. As previously mentioned, ALP is considered an early osteogenic marker [38]. OPN is a calcium binding protein involved in the biomineralization and remodeling of bone tissue. Recent literature also highlights the role of OPN in the quality of collagen fibrils in bone [40]. Indeed, it supports that the altered mechanical properties observed in OPN-deficient bone could be due to diminished levels of collagen and mineral content at the tissue level, as well as to the disorganization of the mineralized collagen fibrils [40]. Importantly, high OPN levels have been related to the late osteogenic differentiation [41]. Real time RT-qPCR studies showed a significant upregulation in the expression of both genes by day 21 in cells cultured on hybrid PCL scaffolds, in comparison to 2D cultured cells. Overall, the results gathered in Figure 4 demonstrate the osteoinductive properties of hybrid PCL scaffolds, which represent a potential alternative matrix to guide the osteogenic differentiation.

## 4. Conclusions

Design of macro/micro porous scaffolds was carried out combining two different technologies in the same fabrication platform. Very similar fabrication results were obtained for different fabrication series, confirming the homogeneity robustness and repeatability of fabrication technique. DSC, FTIR and XRD analyses confirmed that PCL scaffolds had a semicrystalline nature. Scaffolds showed good mechanical properties, including shape memory capacity, and exhibited porous structure with interconnected micro- and macro-porous architecture. Cell culture experiments demonstrated the hybrid PCL scaffolds as a biocompatible platform that enabled cell culture over time. More interestingly, the characteristics of these scaffolds conferred osteoinductive properties, promoting the expression of early and late osteogenic markers in MC-3T3 osteoblasts. Overall, results prove the hybrid PCL scaffolds are a novel platform capable of guiding the osteogenic differentiation.

## Figures and Tables

**Table 1:** Summary of processing conditions of hybrid PCL scaffolds.

Process Parameters	3D Printing	Electrospinning
**Nozzle/Needle size**	0.4 mm	22 G
**Temperature**	110 °C	23 °C
**Speed**	30 mm/s	5 mm/s
**Infill density**	75%	
**Applied voltage**		11 kV
**Flow**	100%	0.15 mL/min
**Offset distance**	0 cm	9 cm

## Data Availability

Data will be made available on request.

## Supplementary Materials

The following supporting information can be downloaded at: https://www.mdpi.com/article/10.3390/pharmaceutics14122843/s1: Figure S1: Homogeneity of Hybrid PCL scaffolds. Figure S2: Optical microscope images of PCL hybrid scaffolds. Figure S3: Cell culture on hybrid PCL scaffolds. Table S1: Bio-Rad PrimePCR primers for real time RT-qPCR.
