# Supplementary material for: Hybrid 3D Printed and Electrospun Multi-Scale Hierarchical Polycaprolactone Scaffolds to Induce Bone Differentiation
Source: Pharmaceutics. 2022 Dec 19;14(12):2843. doi: 10.3390/pharmaceutics14122843 (PMC9781012; doi:10.3390/pharmaceutics14122843)
Supplement: Supplementary file 1 [file pharmaceutics-14-02843-s001.zip › pharmaceutics-2060069-supplementary.pdf]

Table S1. Bio-Rad PrimePCR primers for real time RT-qPCR.

**Gene symbol**	Gene Name	**PrimePCR ID**
Alpl	Alkaline phosphatase	qMmuCID0006482
Spp1	Secreted phosphoprotein 1, Opn	qMmuCED0040763
Gapdh	Glyceraldehyde-3-phosphate dehydrogenase	qMmuCED0027497

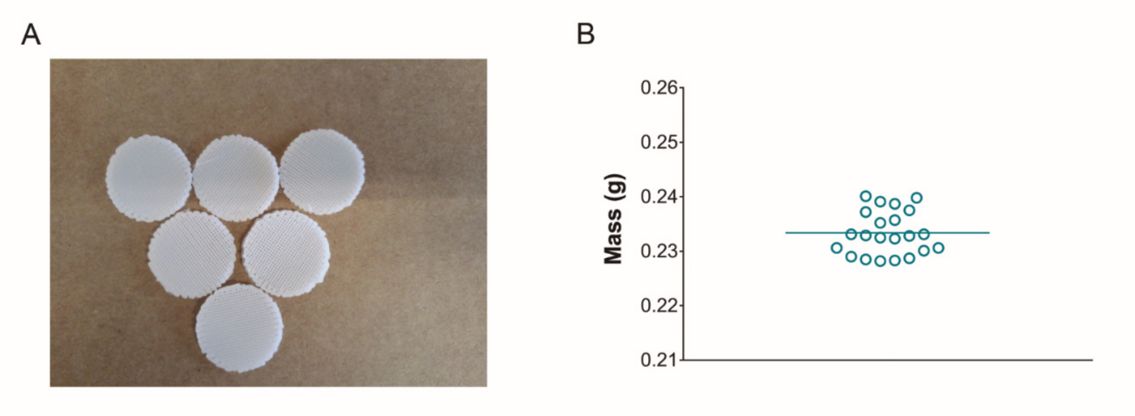

Figure S1. Homogeneity of Hybrid PCL scaffolds. (A) Macroscopic image of Hybrid PCL scaffolds. (B) Mass distribution of Hybrid PCL scaffolds.

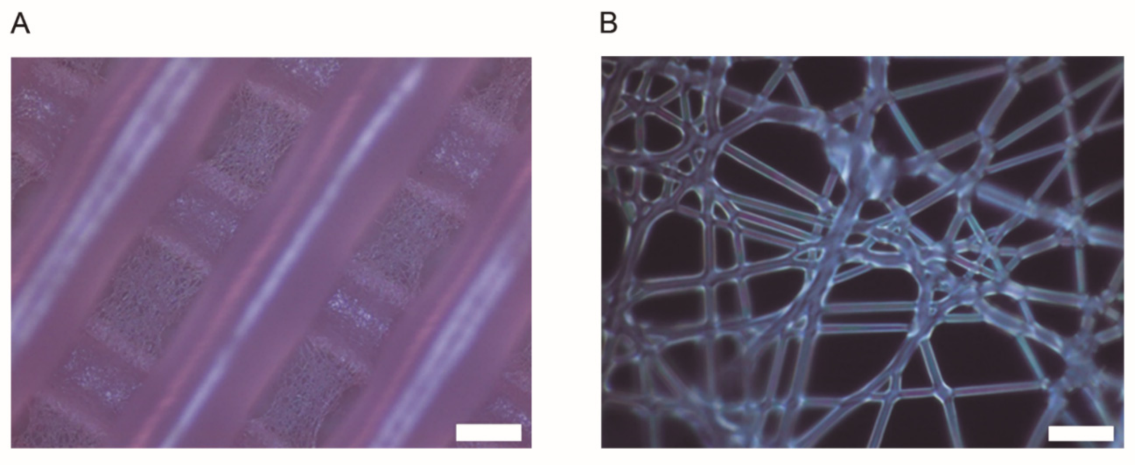

Figure S2. Optical microscope images of PCL hybrid scaffolds. Scale bars: (A) 200 µm and (B) 10 µm.

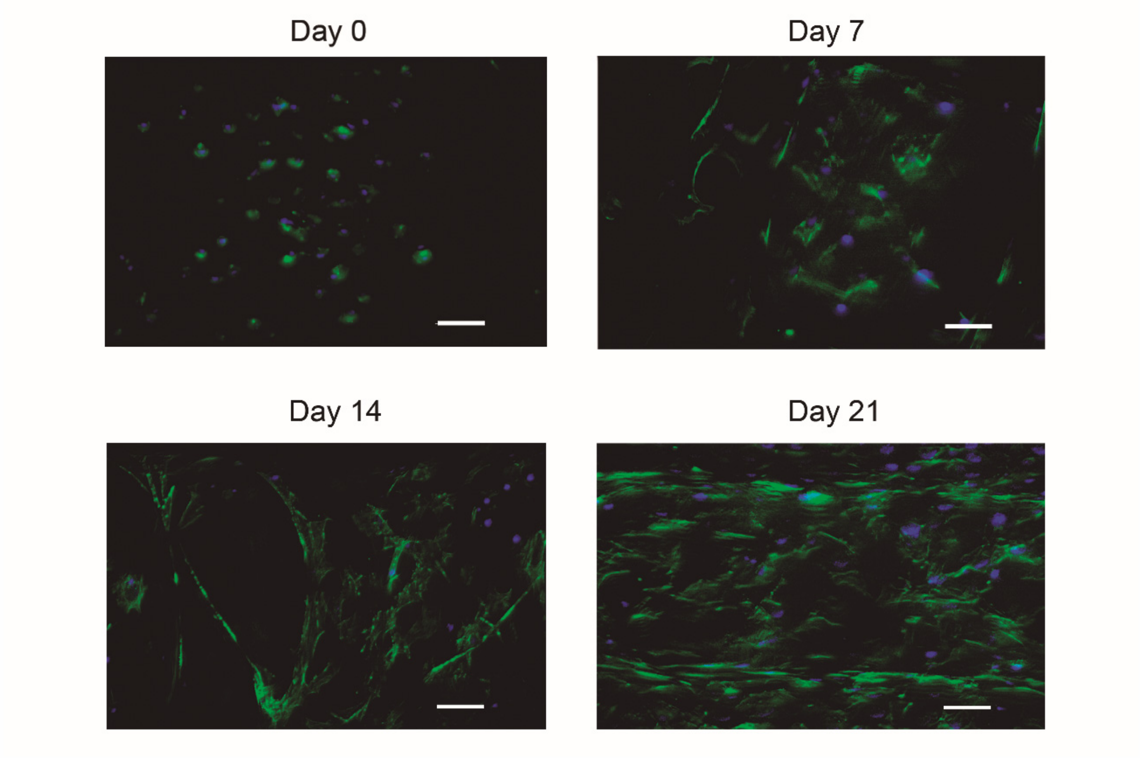

Figure S3. Cell culture on hybrid PCL scaffolds. MC-3T3 murine osteoblast culture on hybrid PCL scaffolds: fluorescent DAPI/Phalloidin micrographs for cell distribution and morphology. Scale bars = 60 μm.
